# Enhancement of Acetone Gas-Sensing Responses of Tapered WO_3_ Nanorods through Sputtering Coating with a Thin SnO_2_ Coverage Layer

**DOI:** 10.3390/nano9060864

**Published:** 2019-06-06

**Authors:** Yuan-Chang Liang, Yu Chao

**Affiliations:** Institute of Materials Engineering, National Taiwan Ocean University, Keelung 20224, Taiwan; h0979620406@gmail.com

**Keywords:** coverage layer, microstructure, sputtering coating, composite nanorods, gas-sensing

## Abstract

WO_3_–SnO_2_ composite nanorods were synthesized by combining hydrothermal growth of tapered tungsten trioxide (WO_3_) nanorods and sputter deposition of thin SnO_2_ layers. Crystalline SnO_2_ coverage layers with thicknesses in the range of 13–34 nm were sputter coated onto WO_3_ nanorods by controlling the sputtering duration of the SnO_2_. The X-ray diffraction (XRD) analysis results demonstrated that crystalline hexagonal WO_3_–tetragonal SnO_2_ composite nanorods were formed. The microstructural analysis revealed that the SnO_2_ coverage layers were in a polycrystalline feature. The elemental distribution analysis revealed that the SnO_2_ thin layers homogeneously covered the surfaces of the hexagonally structured WO_3_ nanorods. The WO_3_–SnO_2_ composite nanorods with the thinnest SnO_2_ coverage layer showed superior gas-sensing response to 100–1000 ppm acetone vapor compared to other composite nanorods investigated in this study. The substantially improved gas-sensing responses to acetone vapor of the hexagonally structured WO_3_ nanorods coated with the SnO_2_ coverage layers are discussed in relation to the thickness of SnO_2_ coverage layers and the core–shell configuration of the WO_3_–SnO_2_ composite nanorods.

## 1. Introduction

Tungsten trioxide (WO_3_) is an n-type wide bandgap semiconductor with various functionalities [1,2,3,4]. Among numerous applications, WO_3_ with various morphologies has received extensive attention as a forward-looking gas-sensing material due to its high sensitivity and stability toward target gases [1,5]. For example, it has been used to detect methane vapor, NO_2_ gas, and CO gas with distinct sensing responses [1,6]. However, WO_3_ has various crystallographic structures [2]. Most gas-sensing properties are reported for the monoclinic structured WO_3_; by contrast, reports for the hexagonal structured WO_3_ being used as gas sensor materials are limited in number.

Oxides in a one-dimensional architecture have the advantage of high sensitivity and fast response/recovery speed due to their high surface-to-volume ratio and great surface activity compared to bulk or thin-film form [7,8,9]. Therefore, the application of one-dimensional WO_3_ nanostructures is one of the main strategies for increasing their gas-sensing performances. Many methods such as hydrothermal and electrospinning techniques have been proposed to fabricate one-dimensional WO_3_ nanostructures [10,11,12]. The hydrothermal synthesis of one-dimensional WO_3_ nanostructures yields a large amount of WO_3_ nanostructures from a solution at process temperatures lower than 250 °C. This method has the advantages of large-scale amount fabrication, low process cost, and easy process parameter control; therefore, hydrothermal methods are promising for synthesizing one-dimensional WO_3_ crystals for gas-sensing applications. Recently, an improved gas-sensing ability of nanostructured WO_3_ was achieved through heterostructure engineering. The intrinsic gas-sensing abilities of nanostructured WO_3_ toward various target gases can be substantially enhanced through the coupling with other semiconductor oxides. For example, microwave synthesized Fe_2_O_3_-decorated WO_3_ nanostructures exhibit improved H_2_S gas-sensing performance [13]. Electrospinning method-derived NiO particles functionalized with WO_3_ porous composites demonstrate enhanced acetone gas-sensing responses [11]. WO_3_ nanosheets loaded with SnO_2_ nanoparticles exhibit enhanced methane-sensing performance. Moreover, it has been shown that the loading content of SnO_2_ nanoparticles has an important influence on the sensing behavior of WO_3_–SnO_2_ nanocomposites [6].

Among the various coupling oxides integrated into WO_3_, SnO_2_ is also an n-type wide bandgap semiconductor, widely used as a gas-sensing material. It has been used to detect methanol, ethanol, and ethylene glycol gases with desirable sensing performance [14,15,16]. Although improvement in the gas-sensing performance of SnO_2_ nanoparticle-decorated monoclinic WO_3_ nanosheets and tetragonal SnO_2_-monoclinic WO_3_ composite films has been reported [4,6], gas-sensing properties of hexagonally structured WO_3_ nanorods coupled with thin coverage layers of SnO_2_ have not yet been proposed. This might hinder the potential applications of hexagonally structured WO_3_-based composite nanorods in gas sensor devices. In this study, SnO_2_ thin layers with various thicknesses were sputter coated onto hexagonally structured WO_3_ nanorods. Sputtering has advantages for in situ growing crystalline oxides with tunable film thickness [17]. The microstructure-dependent gas-sensing behaviors of the hydrothermally derived WO_3_ nanorods sputter coated with thin layers of SnO_2_ were systematically investigated in this study.

## 2. Materials and Methods

For this study, 50 nm thick WO_3_ seed layers were pre-grown on 300 nm thick SiO_2_/Si substrates before the hydrothermal growth of WO_3_ nanorods. A tungsten metallic disc with a diameter of 2 inches was used as the sputtering target to prepare the WO_3_ seed layer. The RF sputtering power of the tungsten target was fixed at 80 W and the seed layer growth temperature was maintained at 500 °C with an Ar/O_2_ ratio of 3:2; the gas pressure during sputter deposition was fixed at 1.33 Pa. Afterwards, the WO_3_ seed layer was annealed at 600 °C for 1 h in ambient air. An amount of 50 mL of deionized water was mixed with 1.65 g sodium tungstate dehydrate powders (Na_2_WO_4_·2H_2_O) for obtaining a precursor solution. The pH value of the precursor solution was regulated by adding oxalic acid and HCl (35%) to pH = 2.45. Subsequently, an amount of 12.5 mL of precursor solution was mixed with 0.175 g sodium chloride and delivered to a 20 mL Teflon-lined autoclave. The hydrothermal growth of WO_3_ nanorods was carried out at 180 °C for 3.5 h by steeping the WO_3_ seed layer-coated substrates in the reaction Teflon-lined autoclave. After the hydrothermal synthesis reaction, the autoclave was cooled down to room temperature. The samples were removed, repeatedly washed with deionized water, and air dried. For the synthesis of WO_3_–SnO_2_ composite nanorods, a tin metallic disc with a diameter of 2 inches was used as the sputtering target to prepare WO_3_–SnO_2_ composite nanorods with various SnO_2_ shell layer thicknesses. The DC sputtering power of the tin target was fixed at 20 W. The growth temperature was maintained at 500 °C with an Ar/O_2_ ratio of 2:1; the gas pressure during sputtering deposition was fixed at 1.33 Pa. The sputtering duration of the SnO_2_ thin films was varied from 10 to 30 min to modulate the sputter-coated SnO_2_ layer thickness on the surfaces of the WO_3_ nanorods. Notably, WS-1, WS-2, and WS-3 represented WO_3_–SnO_2_ composite nanorods prepared with SnO_2_ thin-film sputtering durations of 10, 20, and 30 min, respectively.

The nanorod samples were analyzed by X-ray diffraction (XRD; Bruker D2 PHASER, Karlsruhe, Germany) to investigate their crystal structures. The surface feature of nanorod samples was investigated by scanning electron microscopy (SEM; Hitachi S-4800, Tokyo, Japan). High-resolution transmission electron microscopy (HRTEM) equipped with energy-dispersive X-ray spectroscopy (EDS) (Philips Tecnai F20 G2, Amsterdam, The Netherland) was used to investigate the detailed structure and compositional distribution of the nanorod samples. X-ray photoelectron spectroscopy (XPS; PHI 5000 VersaProbe, Chigasaki, Japan) analysis was used to determine the chemical binding status of the elements in the nanorod samples. Silver electrodes were coated onto the surface of the nanorod samples for gas-sensing measurements. The gas sensors made from various nanorods were placed in a closed vacuum chamber and various concentrations of acetone vapor (100, 250, 500, 750, and 1000 ppm) were introduced into the test chamber, using dry synthetic air as a carrier gas. For the NO_2_, HN_3_, and H_2_ gas-sensing response measurements, the concentration of various target gases was controlled by changing the mixing ratio of the target gas and dry synthetic air. The concentrations of NO_2_, NH_3_, and H_2_ gases were controlled to 5, 100, and 100 ppm, respectively. The gas-sensing response of the gas sensors to reducing gases is defined as Ra/Rg (Rg/Ra for oxidizing gas), where Ra is the gas sensor resistance in the absence of target gas and Rg is the resistance in the target gas.

## 3. Results and Discussion

**Figure 1:**
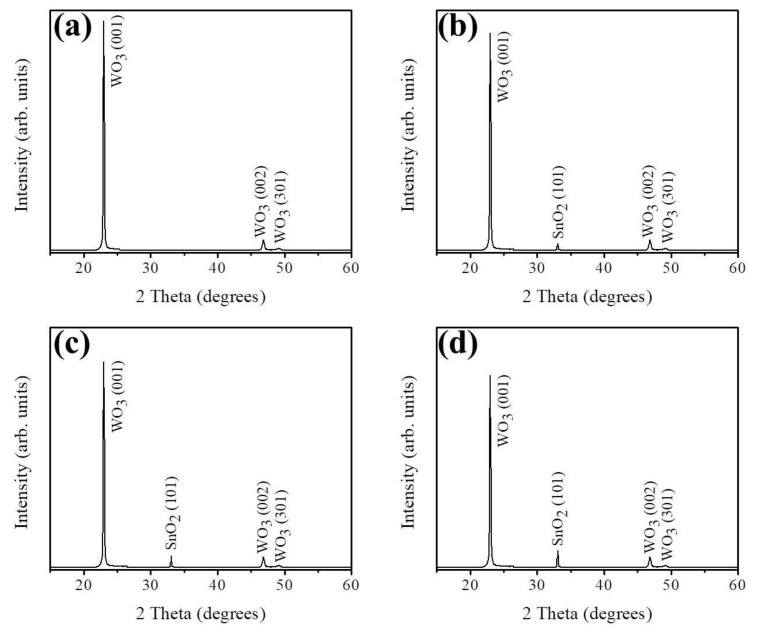
X-ray diffraction (XRD) patterns of various nanorods: (**a**) WO_3_, (**b**) WS-1, (**c**) WS-2, and (**d**) WS-3.

Figure 1a shows the XRD pattern of hydrothermally derived WO_3_ nanorods. The distinct Bragg reflections are ascribed to the (001), (002), and (301) of hexagonal WO_3_ phase according to JCPDS No. 00-033-1387. Noteworthy, the intense (001) Bragg reflection in Figure 1a demonstrated that highly c-axis-oriented WO_3_ crystals were formed. Figure 1b–d shows the XRD patterns of the WO_3_ nanorods sputter coated with various thicknesses of SnO_2_ layers. The corresponding XRD patterns exhibited a visible Bragg reflection centered at approximately 34.1°, which can be ascribed to tetragonal SnO_2_ (101) (JCPDS No. 00-002-1337). Notably, with an increase in the sputtering duration of SnO_2_ thin films, the intensity of the SnO_2_ (101) peak gradually increased, revealing an increase in the thickness of the SnO_2_ layers on the composite nanorods (Figure 1b–d). No trace of other evident Bragg reflections from impurity phase were observed. Obviously, crystalline WO_3_–SnO_2_ composite nanorods with favorable properties were successfully synthesized by sputter-assisted coating of thin layers of SnO_2_ on the WO_3_ nanorods.

**Figure 2:**
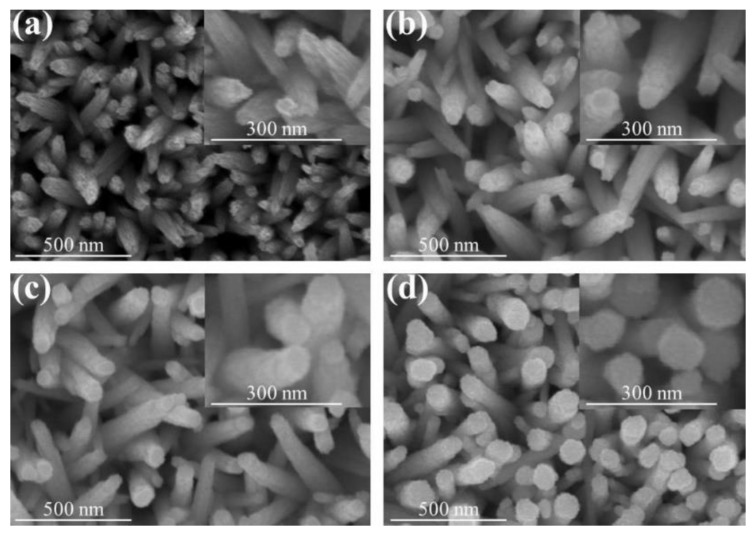
Scanning electron microscopy (SEM) images of various nanorods: (**a**) WO_3_, (**b**) WS-1, (**c**) WS-2, and (**d**) WS-3. The corresponding high magnification images are shown in the insets of the figures.

SEM micrographs and the corresponding high magnification images of as-synthesized WO_3_ nanorods and various WO_3_–SnO_2_ composite nanorods are shown in Figure 2. The micrographs of the WO_3_ nanorods in Figure 2a show that the pristine WO_3_ nanorods feature obvious stripes on their surfaces extending along their growth directions and sharply structured heads. Moreover, the body is conically shaped. Figure 2b–d shows SEM images of WO_3_ nanorods sputter coated with SnO_2_ shell layers with various thicknesses. These WO_3_–SnO_2_ composite nanorods had a different morphology compared with those of the pristine WO_3_ nanorods. Notably, in Figure 2b, the WS-1 composite nanorods had a polycrystalline structure on the surfaces of the WO_3_ nanorods. Further increasing the SnO_2_ sputtering duration, from Figure 2c to Figure 2d, the morphology of the sputter-coated SnO_2_ shell layer gradually changed from tiny particle-feature-coverage layer to the coverage layer consisting of large crystal agglomerates. Notably, the shape of the top region of the composite nanorods in Figure 2d also changed from the original conical pileup to a cylindrical shape and all nanorods took on uniform cylindrical shapes. The SEM images showed that the surface morphology of the WO_3_–SnO_2_ composite nanorods varied with the sputtering duration of the SnO_2_. The roughening of the SnO_2_ coverage layer of the composite nanorods with prolonged sputtering duration was clearly demonstrated herein. The similar phenomenon has also been demonstrated in the ZnO–ZnS composite nanorod system synthesized by sputter-assisted coating of the ZnS shell layer on ZnO nanorods with different sputtering durations [18].

**Figure 3:**
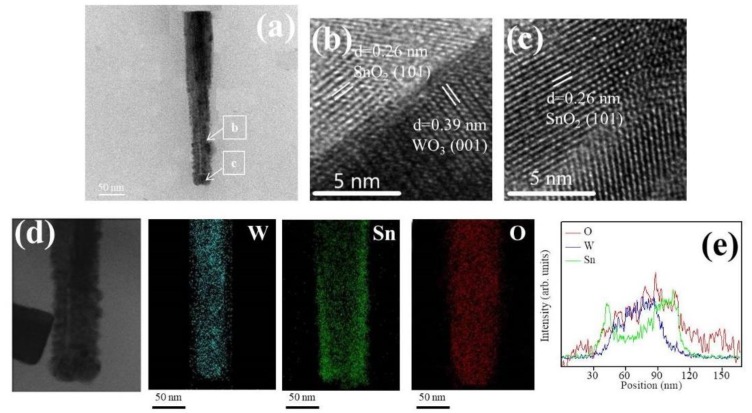
(**a**) Low magnification transmission electron microscopy (TEM) image of a WS-1 nanorod. (**b,c**) High-resolution TEM (HRTEM) images of the nanorod taken from different regions. (**d**) W, Sn, and O elemental mapping images of the nanorod. (**e**) Elemental line-scanning profiles across the nanorod.

The detailed morphology, the SnO_2_ coverage thickness, and the elemental distribution of various WO_3_–SnO_2_ composite nanorods (WS-1, WS-2, and WS-3) were further examined by TEM. Figure 3a shows a low magnification WS-1 nanorod. The composite nanorod exhibited a conical pileup morphology. The coverage layer consisted of tiny particle crystals and the thickness of the coverage layer was estimated to be approximately 13 nm at the top region of the nanorod. Figure 3b,c shows the high-resolution TEM (HRTEM) images of the WS-1 composite nanorod taken from the local interfacial regions of WO_3_/SnO_2_. The lattice fringes with a spacing of 0.39 nm in the inside region of the composite nanorods were assigned to the interplanar distance of hexagonal WO_3_ (001). In addition, the lattice fringes with a spacing of approximately 0.26 nm in the outside region of the composite nanorods were attributed to the interplanar distance of tetragonal SnO_2_ (101). Figure 3d demonstrates the Sn, W, and O elemental mapping images of the WS-1 composite nanorod. The W element was located inside the composite nanorod, revealing the position of the WO_3_ rod template. The Sn element spatially enclosed the whole rod body, demonstrating a homogeneous coverage of the SnO_2_ on the WO_3_ nanorods through sputtering SnO_2_ deposition. Similarly in the cross-sectional EDS line-scanning profiles (Figure 3d), the Sn and O signals were mainly distributed through the whole composite nanorod and the marked W signal was confined to the inner region of the composite nanorod, indicating that the composite nanorod consisted of a WO_3_ core and a SnO_2_ shell coverage layer.

**Figure 4:**
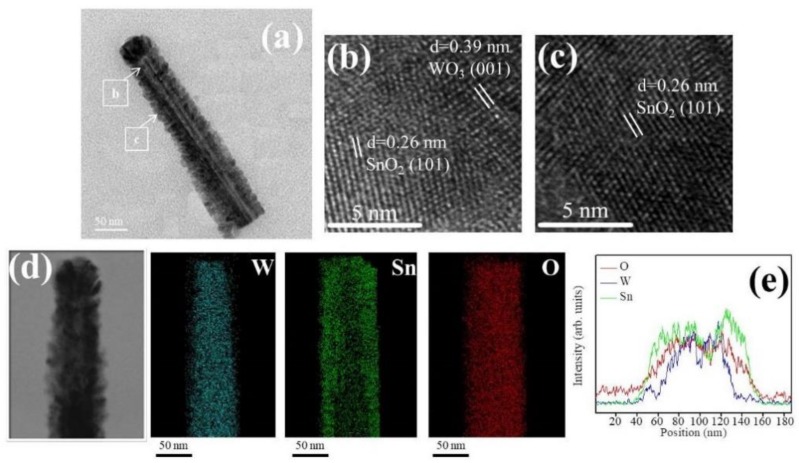
(**a**) Low magnification TEM image of a WS-2 nanorod. (**b**,**c**) HRTEM images of the nanorod taken from different regions. (**d**) W, Sn, and O elemental mapping images of the nanorod. (**e**) Energy-dispersive X-ray spectroscopy (EDS) line-scanning profiles across the nanorod.

**Figure 5:**
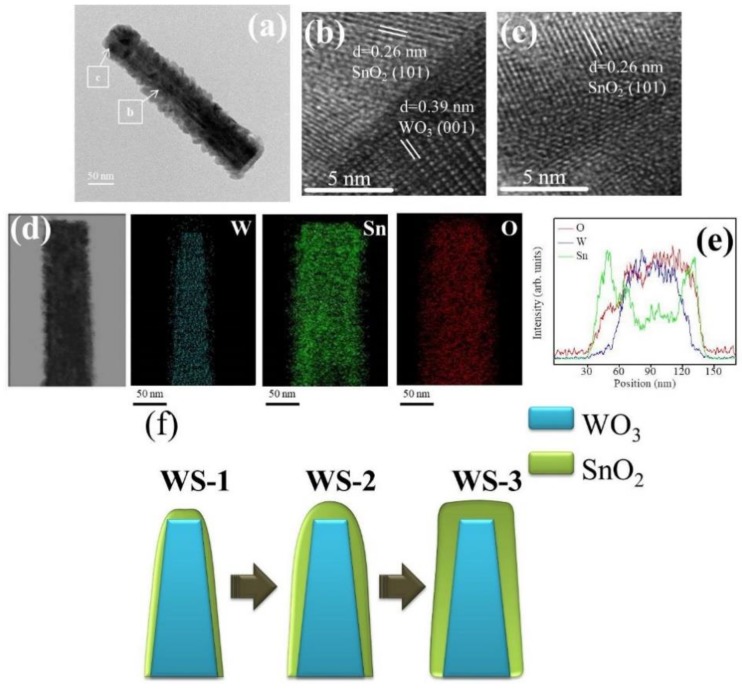
(**a**) Low magnification TEM image of a WS-3 nanorod. (**b**,**c**) HRTEM images of the nanorod taken from different regions. (**d**) W, Sn, and O elemental mapping images of the nanorod. (**e**) EDS line-scanning profiles across the nanorod. (**f**) Schematics of the WS-1, WS-2, and WS-3 nanorods.

Figure 4a shows a low magnification TEM image of the WS-2 nanorod. Similar to the WS-1 nanorod shown in Figure 3a, the SnO_2_ coverage layer of the WS-2 still consisted of tiny SnO_2_ crystals. The SnO_2_ coverage layer, however, was denser and the crystal size of the SnO_2_ was larger than that in WS-1. The morphology of the WS-2 composite nanorod was more cylindrically shaped. The SnO_2_ coverage thickness at the top region of the composite nanorod was evaluated to be approximately 25 nm. Figure 4b,c shows the HRTEM images of the composite nanorods taken from the interfacial regions. The analysis the lattice fringes confirmed the crystal structures of the SnO_2_ coverage layer and WO_3_ nanorod. Furthermore, in Figure 4d, the Sn, W, and O elemental mapping images of the WS-2 composite nanorod revealed the Sn and O elements to be homogeneously distributed over the whole composite nanorod. The W element was confined in the inner regions of the composite nanorod. The EDS line-scanning profiles across the composite nanorod in Figure 4e supported the elemental mapping analysis results that the composite nanorods demonstrated a good WO_3_–SnO_2_ core–shell structure. Figure 5a shows a low magnification image of the WS-3 composite nanorod. The morphology of the WS-3 nanorod took on a fully cylindrical shape after the SnO_3_ sputtering deposition for 30 min. The crystal size of the SnO_2_ coverage layer of the WS-3 nanorod was substantially increased through the prolonged sputtering duration of SnO_2_ in comparison with those of the WS-1 and WS-2 nanorods. Moreover, these large SnO_2_ crystals or aggregates resulted in a rugged surface morphology of the WS-3 nanorods. The SnO_2_ coverage layer thickness at the top region of the composite nanorod was approximately 34 nm. The arrangements of local lattice fringes of the SnO_2_ coverage layer and of the WO_3_ core were also characterized in the HRTEM images (Figure 5b,c). Figure 5d,e demonstrates the homogeneous surface coverage of the Sn element through the whole WO_3_ nanorod. Notably, the intensity of the Sn signal in the EDS line-scanning profiles of various WO_3_–SnO_2_ composite nanorods increased with SnO_2_ sputtering duration as exhibited in the corresponding EDS spectra profiles, revealing an increased thickness of the SnO_2_ coverage layers on the composite nanorods. The TEM results herein revealed a good coverage of the sputter-deposited SnO_2_ thin films on the surface of the WO_3_ nanorods. A schematic summary of the morphology changes of the WO_3_–SnO_2_ composite nanorods prepared with various sputtering durations of SnO_2_ based on the TEM analysis results are also shown in Figure 5f.

**Figure 6:**
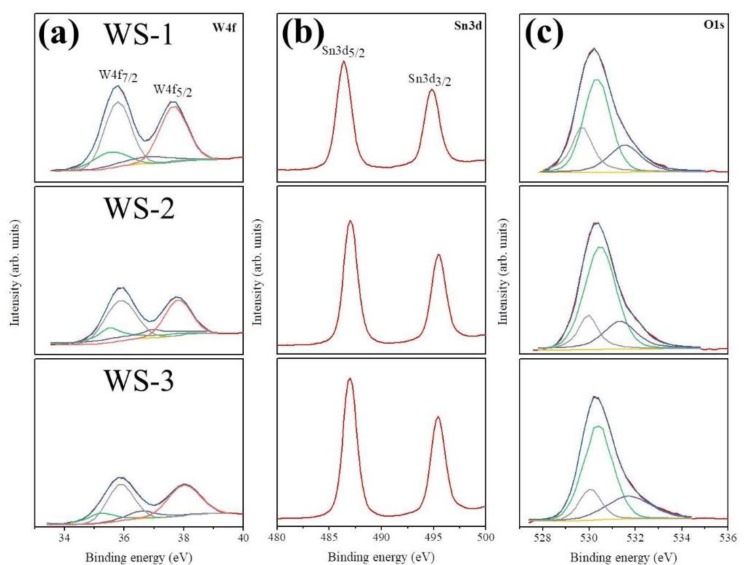
High resolution X-ray photoelectron spectroscopy (XPS) spectra of various WO_3_–SnO_2_ composite nanorods: (**a**) W4f core-level doublet, (**b**) Sn3d core-level doublet, and (**c**) O1s peak.

The peak intensity of W4f core-level doublets originating from the WO_3_ nanorods decreased and the peak intensity of the Sn 3d core-level doublets of the sputtered SnO_2_ coverage layers increased with the increase of the sputtering duration of SnO_2_ in Figure 6a,b. This reveals that the variation of the SnO_2_ shell layer thickness on the composite nanorods is controlled by the change in the sputtering duration of the SnO_2_ thin films. Figure 6a displays the W4f_7/2_ and W4f_5/2_ peaks centered at 35.9 and 37.9 eV, respectively, for the WO_3_ nanorods coated with various thicknesses of SnO_2_ thin films. The Gaussian deconvolution results of the W4f spectra of the various composite nanorods illustrated the contributions corresponding to the W^5+^ and W^6+^ states in the WO_3_. The main peaks centered at 35.9 and 37.9 eV correspond to W^6+^ binding states; moreover, the weaker intensity and lower binding energies for the subpeaks centered at 35.2 and 36.5 eV correspond to W^5+^ binding states. Figure 6b displays XPS Sn3d spectra of various WO_3_–SnO_2_ composite nanorods. A correspondence Sn3d_5/2_ peak centered at 487.1 eV and a Sn3d_3/2_ peak centered at approximately 495.4 eV were observed. The binding energy difference between the Sn3d_5/2_ and Sn3d_3/2_ corresponded to the chemical binding component of Sn^4+^ in the SnO_2_ [15,17]. The O1s XPS spectra of various composite nanorods in Figure 6c demonstrated three subcomponents. The component with a binding energy of approximately 529.8 eV is assigned to the tungsten oxide that formed the W–O bonds [19]. The second component with a binding energy of 530.5 eV is assigned to the tin oxide that formed the strong Sn–O bonds [15,17], and the small degree of oxygen vacancies and/or oxygen species chemisorbed from the ambient air were demonstrated in the component centered at approximately 531.9 eV.

**Figure 7:**
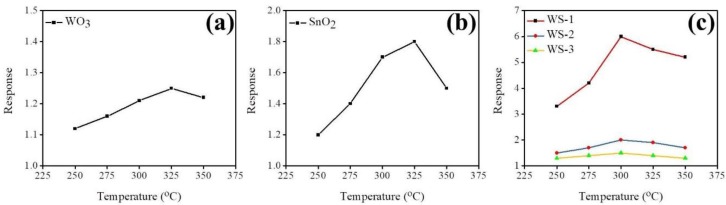
Temperature-dependent gas-sensing responses for the gas sensors on exposure to 100 ppm acetone vapor at operating temperatures ranging from 250 to 350 °C: (**a**) WO_3_ nanorods, (**b**) SnO_2_ thin film, (**c**) various composite nanorods: WS-1 (red line), WS-2 (blue line), and WS-3 (yellow line).

Figure 7a–c demonstrates the variation of the temperature-dependent gas-sensing responses of the pristine WO_3_ nanorods, a 50 nm thick SnO_2_ film, and various WO_3_–SnO_2_ composite nanorods upon exposure to 100 ppm acetone vapor. The optimum operating temperature of the composite nanorods is lower than that of the WO_3_ nanorods and SnO_2_ thin film, in which they exhibited an optimal operating temperature of 325 °C. Previously, Zhang et al. showed that the La_2_O_3_-decorated SnO_2_ gas sensors feature a 2-fold higher gas-sensing performance improvement at 250 °C, which is lower than the optimum operating temperature of pristine SnO_2_ of 300 °C [20]. A decreased operating temperature of the oxide semiconductor through a heterostructure structure has also been shown in the ZnO–SnO_2_ system [21]. The optimal operating temperature for acetone gas-sensing examinations of various WO_3_–SnO_2_ composite nanorods was chosen to be 300 °C for this study.

**Figure 8:**
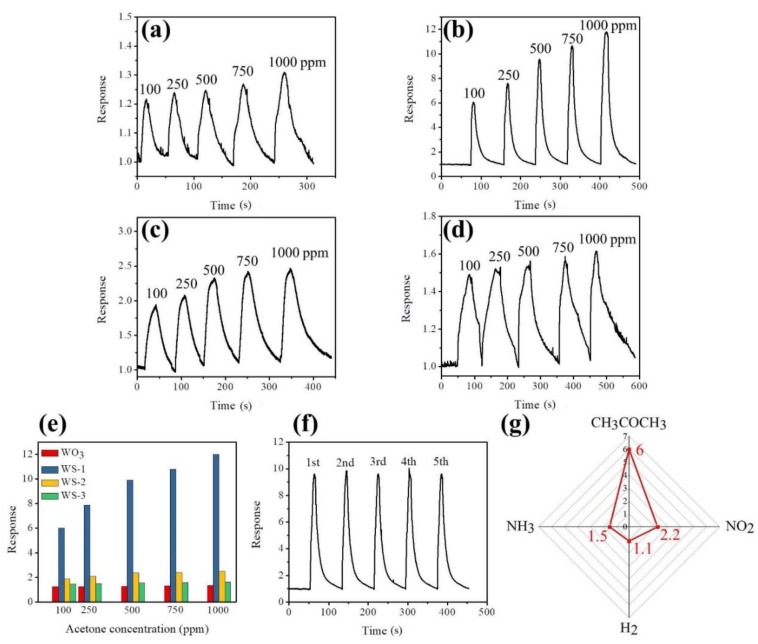
Dynamic gas-sensing response–recovery curves of various nanorods upon exposure to different acetone vapor concentrations (100 to 1000 ppm): (**a**) WO_3_, (**b**) WS-1, (**c**) WS-2, and (**d**) WS-3 nanorods. (**e**) Gas-sensing response values vs. acetone vapor concentration for various nanorods. (**f**) Cyclic gas-sensing response curves for the WS-1 nanorods exposed to 500 ppm acetone vapor. (**g**) Gas-sensing selectivity of the WS-1 nanorods exposed to 100 ppm of CH_3_COCH_3_, NH_3_, and H_2_, and 5 ppm of NO_2_.

Figure 8a–d shows the dynamic acetone gas-sensing response of pristine WO_3_ nanorods and various WO_3_–SnO_3_ nanorods upon exposure to various acetone vapor concentrations. The acetone gas-sensing responses of gas sensors made from various nanorods increased with the acetone vapor concentration, revealing that an increased number of acetone molecule numbers interacted with the absorbed oxygen species on the surfaces of the nanorods [15]. A plot of the acetone gas-sensing response vs. acetone vapor concentration is shown in Figure 8e. The acetone gas-sensing response of the pristine WO_3_ nanorods on exposure to 100–1000 ppm acetone vapor ranged from 1.25 to 1.35. The smooth and distinct dynamic response curves on exposure to various acetone vapor concentrations are demonstrated for the WO_3_ nanorods in Figure 8a, revealing that the WO_3_ nanorods are responding to acetone vapor; however, the response values are not high enough for practical use. By contrast, in Figure 8e, the WO_3_ nanorods coated with various thicknesses of SnO_2_ coverage layers exhibit an improved acetone gas-sensing capability compared with the pristine WO_3_ nanorods. The highest level of enhancement in acetone gas-sensing response was observed for the WS-1 nanorods. Their responses ranged from 6.3 to 12.1 upon exposure to 100 to 1000 ppm acetone vapor, respectively. An approximately 5-fold increase in the acetone gas-sensing response upon exposure to 100 ppm acetone vapor was observed for WO_3_ nanorods coated with 13 nm thick SnO_2_ films. Notably, thicker SnO_2_ coatings on the WO_3_ nanorods did not further enhance the acetone gas-sensing response of the composite nanorods. The acetone gas-sensing response decreases with SnO_2_ layer thickness as shown in Figure 8e. The optimal coating thickness of the SnO_2_ thin layer is approximately 13 nm for the WO_3_–SnO_2_ composite nanorod system herein. The response and recovery times for the gas sensors made from various nanorod samples are defined as the duration required to drop the 90% resistance on exposure to the target gas and that to increase 90% resistance with the removal of the target gas. The response times for the pristine WO_3_ nanorods exposed to 100–1000 ppm acetone vapor concentrations ranged from 8 to 17 s, whereas recovery times ranged from 33 to 58 s in the same acetone vapor concentration range. By contrast, the response times for the sensors made from the WS-2 and WS-3 composite nanorods ranged from 6 to 16 s and 20 to 32 s, respectively. The recovery times of the WS-2 and WS-3 composite nanorods were 38–83 s and 35–100 s in the acetone concentration range of 100–1000 ppm, respectively. Substantially increased response and recovery times were observed for the composite nanorods with the thickest SnO_2_ coverage layer. Notably, the response times and recovery times for the WS-1 composite nanorods in the same test acetone vapor concentration range ranged between 3–13 s and 28–51 s, respectively. The slightly improved response and recovery speeds together with the substantial enhancement in gas-sensing responses revealed an improved acetone gas-sensing performance of the WO_3_–SnO_2_ composite nanorods with an optimal SnO_2_ thin layer thickness of approximately 13 nm. Figure 8f displays cyclic acetone gas-sensing tests for the WS-1 composite nanorods exposed to 500 ppm acetone vapor. Steady gas-sensing activity under five test cycles was observed, confirming that WS-1 nanorods were reproducible and stable for detecting acetone vapor. Figure 8g shows the gas-sensing selectivity of CH_3_COCH_3_, H_2_, and NH_3_ gases with concentrations of 100 and 10 ppm for NO_2_ gas for the WS-1 composite nanorods. The WS-1 composite nanorods exhibited the best gas-sensing response toward acetone vapor among the various target gases. Table 1 summarizes the acetone gas-sensing responses of the WO_3_-based composites operating at the temperature range of 280–400 °C [22,23,24,25,26,27]. In comparison, the WS-1 composite nanorods presented the best acetone vapor detection performance among various reported works.

**Figure 9:**
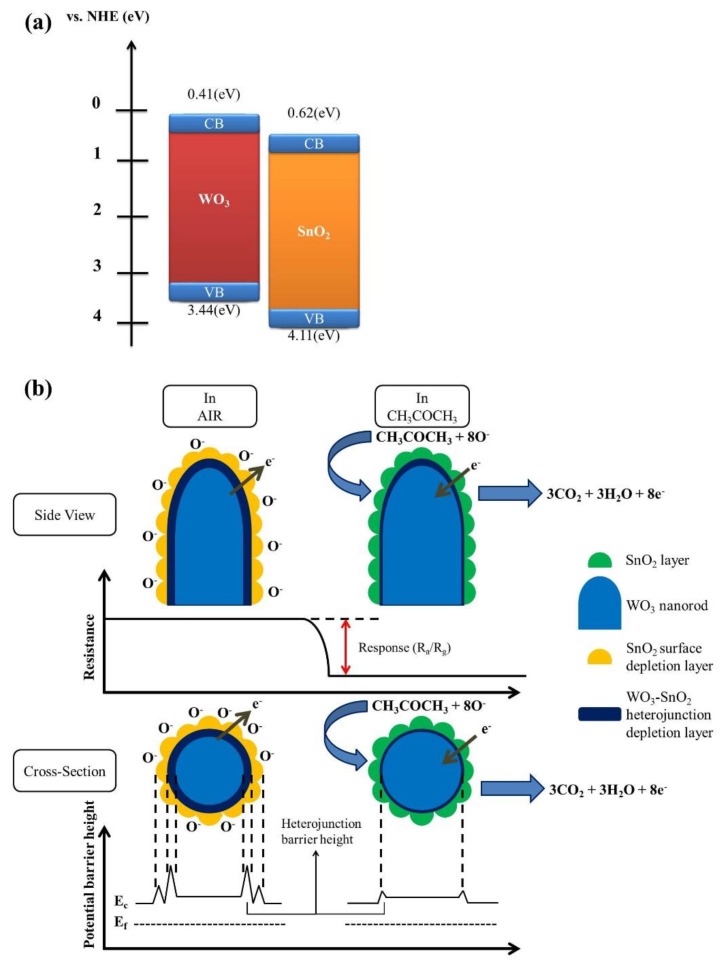
(**a**) The conduction band-edge and valence band-edge positions of the WO_3_ and SnO_2_. (**b**) Schematics of possible gas-sensing mechanism of WS-1 nanorods on exposure to acetone vapor.

The acetone gas-sensing mechanism of WO_3_–SnO_2_ composite nanorods with an optimum thickness of SnO_2_ layer is illustrated in Figure 9. In our assessment of the gas-sensing mechanism, we follow the approach outlined in reference [28]. When thin SnO_2_ coverage layers are sputtered onto the surfaces of the WO_3_ nanorods, an interfacial depletion layer forms as a WO_3_/SnO_2_ heterojunction develops. According to the band alignment structure of the WO_3_/SnO_2_ in Figure 9a [29,30], an electron depletion layer initially forms on the WO_3_ side. Upon exposure of the WO_3_–SnO_2_ composite nanorods to ambient air, oxygen molecules remove surface electrons from the composite nanorods, thus forming adsorbed negatively charged oxygen species (Figure 9b). In this way, the sputter-coated SnO_2_ thin layers in the composite nanorods become partially depleted at some degrees under the sensor operating conditions. Notably, the extent of this depletion is highly dependent on the sensor operating temperature. The thickness of the surface depletion layer of the SnO_2_ at 327 °C in ambient air has been shown to be approximately 21 nm [31]. In ZnO–SnO_2_ core–shell nanowires, it has been proposed that the SnO_2_ shell layers with a thickness of 15–20 nm was fully depleted in the range of sensor operating temperatures of 200–400 °C [32]. The complete depletion of the SnO_2_ shell layer significantly affects the variation of the electron depletion layer width at the ZnO/SnO_2_ heterointerface during gas exposure, thus leading to a substantially enhanced gas-sensing performance [30]. In the current work, the extraction of surface electrons widened the thickness of the interfacial depletion layer and thus increased the interfacial potential barrier. In this way, the width of the electron conduction path through the single crystalline WO_3_ nanorods is reduced and the resistance of the composite nanorods is increased (Figure 9b). When acetone vapor is introduced into the test chamber, the interaction between the reducing acetone vapor and the adsorbed oxygen species of the surfaces of the composite nanorods can be expressed as follows [33]:

CH_3_COCH_3_ (gas) + 8O^−^_ads_ → 3CO_2_ + 3H_2_O + 8e^−^. (1)

The release of free electrons from the adsorbed surface oxygen species eliminates the surface depletion regions in the SnO_2_ coverage layers and further narrows the size of interfacial depletion regions, thus causing the substantial drop in resistance of the composite nanorods. The marked resistance variation of the composite nanorods before and after introducing acetone vapor results in a distinct gas-sensing response of the composite nanorods upon exposure to acetone vapor. The efficiency of this process is critically dependent on the thickness of the SnO_2_ coverage layers. Our work suggests that in WO_3_–SnO_2_ composite nanorods this optimum thickness is close to 13 nm. In its order of magnitude, this value agrees with the results of previous investigations into core–shell SnO_2_–ZnO nanowires [34] and SnO_2_–ZnO nanofibers [35]. It appears that in all cases a substantial improvement in gas response is obtained when the thickness of the coverage layers is close to the respective Debye length.

## 4. Conclusions

WO_3_–SnO_2_ composite nanorods were synthesized by sputter coating a SnO_2_ coverage layer onto the surfaces of WO_3_ nanorods. The sputtering duration of SnO_2_ was varied to control the SnO_2_ coverage layer thickness on the WO_3_ nanorods in the range of 13–34 nm. Crystalline WO_3_–SnO_2_ composite nanorods were formed. Moreover, the evolution of the microstructure of the SnO_2_ coverage layers was characterized as a function of their thickness. Thicker SnO_2_ coverage layers exhibited larger surface crystals or aggregates. The acetone gas-sensing tests revealed that WO_3_–SnO_2_ composite nanorods with a 13 nm thick SnO_2_ coverage layer exhibited the best gas-sensing responses among the various composite nanorods. An optimum match of the SnO_2_ coverage layer thickness with the Debye length inside the coverage layers at the optimum operating temperature range is likely to account for the observed superior acetone gas-sensing responses of the WS-1 composite nanorods.

## Figures and Tables

**Table 1:** Summary of the acetone gas-sensing performances of various WO_3_-based composites operating in the temperature range of 280–400 °C [22,23,24,25,26,27]. N/A, Not available.

Composites	Morphology	Synthesis Method	Operating Temperature (°C)	Acetone Concentration (ppm)	Response (R_a_/R_g_)	Response/Recovery Time (s)
WO_3_-Co_3_O_4_	Rod	Hydrothermal	280	100	5.3	N/A
WO_3_-Cr_2_O_3_	Rod	Thermal evaporation and spin coating	300	200	4	N/A
WO_3_-NiO	Rod	Thermal evaporation and hydrothermal	300	100	3.3	52/60
WO_3_–ZnO	Rod	Hydrothermal	400	300	5	N/A
WO_3_–Rh_2_O_3_	Fiber	Electrospinning	300	1	3.2	N/A
WO_3_–SnO_2_	Fiber	Electrospinning	280	10	3	32/364
WO_3_–SnO_2_ (this work)	Rod	Hydrothermal and sputtering	300	100	6	3/28

## References

[1] Zhao S., Shen Y., Zhou P., Li G., Han C., Wei D., Zhong X., Zhang Y., Ao Y. (2019). Influence of synthesis conditions on microstructure and NO_2_ sensing properties of WO_3_ porous films synthesized by non-hydrolytic sol-gel method. Nanomaterials.

[2] Liang Y.C., Chang C.W. (2019). Preparation of orthorhombic WO_3_ thin films and their crystal quality-dependent dye photodegradation ability. Coatings.

[3] Zhang G., Lu K., Zhang X., Yuan W., Ning H., Tao R., Liu X., Yao R., Peng J. (2018). Enhanced transmittance modulation of SiO_2_-doped crystalline WO_3_ films prepared from a polyethylene oxide (PEO) template. Coatings.

[4] Liang Y.C., Chao Y. (2019). Crystal phase content-dependent functionality of dual phase SnO_2_-WO_3_ nanocomposite films via cosputtering crystal growth. RSC Adv..

[5] Wu C.-S. (2015). Hydrothermal fabrication of WO_3_ hierarchical architectures: Structure, Growth and Response. Nanomaterials.

[6] Xue D., Wang J., Wang Y., Sun G., Cao J., Bala H., Zhang Z. (2019). Enhanced methane sensing properties of WO_3_ nanosheets with dominant exposed (200) facet via loading of SnO_2_ nanoparticles. Nanomaterials.

[7] Liang Y.C., Xu N.C. (2018). Synthesis of TiO_2_-ZnS nanocomposites via sacrificial template sulfidation and their ethanol gas-sensing performance. RSC Adv..

[8] Liang Y.C., Xu N.C., Wang C.C., Wei D.H. (2017). Fabrication of nanosized island-like CdO crystallites-decorated TiO_2_ rod nanocomposites via a combinational methodology and their low-concentration NO_2_ gas-sensing behavior. Materials.

[9] Liang Y.C., Lin T.Y., Lee C.M. (2015). Crystal growth and shell layer crystal-feature-dependent sensing and photoactivity performance of zinc oxide-indium oxide core-shell nanorod heterostructures. CrystEngComm.

[10] Khan M.E., Khan M.M., Cho M.H. (2016). Fabrication of WO_3_ nanorods on graphene nanosheets for improved visible light-induced photocapacitive and photocatalytic performance. RSC Adv..

[11] Zhang J., Lu H., Liu C., Chen C., Xin X. (2017). Porous NiO-WO_3_ heterojunction nanofibers fabricated by electrospinning with enhanced gas sensing properties. RSC Adv..

[12] Wang B., Man W., Yu H., Li Y., Zheng F. (2018). Fabrication of Mo-doped WO_3_ nanorod arrays on FTO substrate with enhanced electrochromic properties. Materials.

[13] Yin L., Chen D., Feng M., Ge L., Yang D., Song Z., Fan B., Zhang R., Shao G. (2015). Hierarchical Fe_2_O_3_@WO_3_ nanostructures with ultrahigh specific surface areas: Microwaveassisted synthesis and enhanced H_2_S-sensing performance. RSC Adv..

[14] Zhao Y., Li Y., Ren X., Gao F., Zhao H. (2017). The effect of Eu doping on microstructure, morphology and methanal-sensing performance of highly ordered SnO_2_ nanorods array. Nanomaterials.

[15] Liang Y.C., Lee C.M., Lo Y.J. (2017). Reducing gas-sensing performance of Ce-doped SnO_2_ thin films through a cosputtering method. RSC Adv..

[16] Wan W., Li Y., Ren X., Zhao Y., Gao F., Zhao H. (2018). 2D SnO_2_ nanosheets: Synthesis, characterization, structures, and excellent sensing performance to ethylene glycol. Nanomaterials.

[17] Liang Y.C., Lo Y.J. (2017). High-temperature solid-state reaction induced structure modifications and associated photoactivity and gas-sensing performance of binary oxide one-dimensional composite system. RSC Adv..

[18] Liang Y.C., Lo Y.R., Wang C.C., Xu N.C. (2018). Shell layer thickness-dependent photocatalytic activity of sputtering synthesized hexagonally structured ZnO-ZnS composite nanorods. Materials.

[19] Liang Y.C., Chang C.W. (2019). Improvement of ethanol gas-sensing responses of ZnO-WO_3_ composite nanorods through annealing induced local phase transformation. Nanomaterials.

[20] Zhang G., Zhang S., Yang L., Zou Z., Zeng D., Xie C. (2013). La_2_O_3_-sensitized SnO_2_ nanocrystalline porous film gas sensors and sensing mechanism toward formaldehyde. Sens. Actuators B.

[21] Ma X., Song H., Guan C. (2013). Enhanced ethanol sensing properties of ZnO-doped porous SnO_2_ hollow nanospheres. Sens. Actuators B.

[22] Zhao X., Ji H., Jia Q., Wang M. (2015). A nanoscale Co_3_O_4_-WO_3_ p-n junction sensor with enhanced acetone responsivity. J. Mater. Sci. Mater. Electron..

[23] Choi S., Bonyani M., Sun G.J., Lee J.K., Hyun S.K., Lee C. (2018). Cr_2_O_3_ nanoparticle-functionalized WO_3_ nanorods for ethanol gas sensors. Appl. Surf. Sci..

[24] Choi S., Lee J.K., Lee W.S., Lee C., Lee W.I. (2017). Acetone sensing of multi-networked WO_3_-NiO core-shell nanorod sensors. J. Korean Phys. Soc..

[25] Dien N.D. (2016). ZnO Microrods surface-decorated by WO_3_ nanorods for enhancing NH_3_ gas sensing performance. J. Sci. Technol..

[26] Kim N.H., Choi S.J., Kim S.J., Cho H.J., Jang J.S., Koo W.T., Kim M., Kim I.D. (2016). Highly sensitive and selective acetone sensing performance of WO_3_ nanofibers functionalized by Rh_2_O_3_ nanoparticles. Sens. Actuators B.

[27] Li F., Gao X., Wang R., Zhang T. (2018). Design of WO_3_-SnO_2_ core-shell nanofibers and their enhanced gas sensing performance based on different work function. Appl. Surf. Sci..

[28] Sun P., Cai Y., Du S., Xu X., You L., Ma J., Liu F., Liang X., Sun Y., Lu G. (2013). Hierarchical α-Fe_2_O_3_/SnO_2_ semiconductor composites: Hydrothermal synthesis and gas sensing properties. Sens. Actuators B.

[29] Aslam I., Cao C., Tanveer M., Khan W.S., Tahir M., Abid M., Idrees F., Butt F.K., Ali Z., Mahmood N. (2014). The synergistic effect between WO_3_ and g-C_3_N_4_ towards efficient visible-light-driven photocatalytic performance. New J. Chem..

[30] Baek J.H., Kim B.J., Han G.S., Hwang S.W., Kim D.R., Cho I.S., Jung H.S. (2017). BiVO_4_/WO_3_/SnO_2_ double-heterojunction photoanode with enhanced charge separation and visible-transparency for bias-free solar water-splitting with a perovskite solar cell. ACS Appl. Mater. Interfaces.

[31] Barsan N., Weimar U. (2001). Conduction model of metal oxide gas sensors. J. Electroceram..

[32] Hwang I.S., Kim S.J., Choi J.K., Choi J., Ji H., Kim G.T., Cao G., Lee J.H. (2010). Synthesis and gas sensing characteristics of highly crystalline ZnO-SnO_2_ core-shell nanowires. Sens. Actuators B.

[33] Yin M., Yao Y., Fan H., Liu S. (2018). WO_3_-SnO_2_ nanosheet composites: Hydrothermal synthesis and gas sensing mechanism. J. Alloys Compd..

[34] Choi S.W., Katoch A., Sun G.J., Kim J.H., Kim S.H., Kim S.S. (2014). Dual functional sensing mechanism in SnO_2_-ZnO core-shell nanowires. ACS Appl. Mater. Interfaces.

[35] Katoch A., Choi S.W., Sun G.J., Kim S.S. (2013). An approach to detecting a reducing gas by radial modulation of electron-depleted shells in core-shell nanofibers. J. Mater. Chem. A.

